# Revisiting the Tenascins: Exploitable as Cancer Targets?

**DOI:** 10.3389/fonc.2022.908247

**Published:** 2022-06-17

**Authors:** Richard P. Tucker, Martin Degen

**Affiliations:** ^1^ Department of Cell Biology and Human Anatomy, University of California, Davis, Davis, CA, United States; ^2^ Laboratory for Oral Molecular Biology, Department of Orthodontics and Dentofacial Orthopedics, University of Bern, Bern, Switzerland

**Keywords:** tenascins, extracellular matrix, tumor stroma, biomarker, anti-cancer therapy

## Abstract

For their full manifestation, tumors require support from the surrounding tumor microenvironment (TME), which includes a specific extracellular matrix (ECM), vasculature, and a variety of non-malignant host cells. Together, these components form a tumor-permissive niche that significantly differs from physiological conditions. While the TME helps to promote tumor progression, its special composition also provides potential targets for anti-cancer therapy. Targeting tumor-specific ECM molecules and stromal cells or disrupting aberrant mesenchyme-cancer communications might normalize the TME and improve cancer treatment outcome. The tenascins are a family of large, multifunctional extracellular glycoproteins consisting of four members. Although each have been described to be expressed in the ECM surrounding cancer cells, tenascin-C and tenascin-W are currently the most promising candidates for exploitability and clinical use as they are highly expressed in various tumor stroma with relatively low abundance in healthy tissues. Here, we review what is known about expression of all four tenascin family members in tumors, followed by a more thorough discussion on tenascin-C and tenascin-W focusing on their oncogenic functions and their potential as diagnostic and/or targetable molecules for anti-cancer treatment purposes.

## 1 Introduction

The extracellular matrix (ECM) is a complex meshwork of various cell-secreted macromolecules that regulates the development and homeostasis of tissues. In humans, the ECM is broadly composed of a mixture of proteins, polysaccharides, and water that, depending on the specific composition, reflect the physiological requirements for a given tissue (1). The main building blocks of the ECM are (i) collagens, (ii) glycoproteins, (iii) proteoglycans, and (iv) elastin. Collagens include the most abundant fibrous proteins of our body. They maintain the structural integrity of tissues by providing tensile strength. Glycoproteins are responsible for either linking different ECM molecules or mediating cell-ECM interactions, while proteoglycans include gel-like elements within the ECMs that allow a tissue to resist compressive forces. Finally, elastin, as its name indicates, is responsible for tissue elasticity (2). The human genome harbors 274 genes encoding for the proteins of the core matrisome (3). Although typical ECM-like domains were already present in the proteins of unicellular organisms, the complexity and diversity of the matrisome mainly arose during the transition from unicellular (protozoa) to multicellular (metazoa) animals. The emergence of more complex and multicellular organisms required major evolutionary innovations, which were accompanied by the creation of more specialized and sophisticated ECM frameworks. Extensive elaborations of ECM proteins by exon shuffling, multiplications, and gene family expansions provided the rich repertoire of ECM molecules required for building the various structures (4, 5).

Initially, the ECM was mainly perceived as an inert physical scaffold for cells. However, this view has changed considerably during recent decades. Not only does the ECM provide structural and mechanical support for tissue integrity, but it is also responsible for the mobilization and availability of growth factors and cytokines (4). Additionally, the three-dimensional (3D) network provides specific biochemical and biomechanical properties that are sensed by specialized transmembrane receptors, such as integrins, syndecans and discoidin domain receptors, which can bind specific motifs in ECM proteins. Receptor ligation results in its activation, triggering signaling cascades that regulate various vital cellular processes, including cell adhesion, migration, proliferation, survival, and differentiation. Moreover, the ECM is subject to specific modifications and remodeling, which tailors the ECM to the unique needs of tissues. Therefore, each tissue is surrounded by an ECM that is fine-tuned in its composition and arrangement, providing the necessary cues for tissue homeostasis.

Since tissue homeostasis is often disrupted in cancers, the ECM surrounding cancer cells is significantly different from the one found in healthy conditions (6, 7). The role of the tumor microenvironment (TME) on cancer cells has only recently begun to be appreciated. Earlier cancer research was focused on the individual cancer cell, oncogenes and tumor suppressors; novel mutations and hyperactive signaling pathways were then identified and exploited as anti-cancer therapies. While this approach revolutionized cancer treatment, it seems clear today that cancers need to be viewed as complex heterogeneous tissues. Apart from cancer cells, cancers also depend on supporting components present in the TME that are not malignant themselves. These include the vasculature, immune cells, fibroblasts, and the ECM. Cellular sources of this tumor-specific ECM might be tumor cells themselves, but more often this matrix is derived from cancer-associated fibroblasts (CAFs). CAFs are believed to evolve from the activation of quiescent fibroblasts by epithelial-mesenchymal interactions, often involving cancer cell-derived growth factors (8). Hence, while carcinoma growth is initiated by transformed epithelial cells, cancers continuously tailor and orchestrate their microenvironment for their specific needs. Consequently, a unique composition and mix of ECM molecules is created, which provides feedback to malignant cells allowing the modulation of various hallmarks of cancers (9–11).

There is mounting evidence that the tumor ECM plays an active role in driving malignancy (9). Hence, novel anti-cancer drugs might be more powerful if they not only aim to eliminate cancers cells, but also try to normalize the TME. Such an approach requires a better understanding of the aberrant tumor cell-stroma crosstalk as well as a better understanding of the tumor-specific ECM molecules that are present. At least two members of the tenascin family of ECM glycoproteins, tenascin-C (TN-C) and tenascin-W (TN-W), are highly expressed in most solid cancers, but are expressed at much lower levels in normal tissues. Both of these tenascins provide multifunctional pro-tumorigenic activities (12). Therefore, tenascins, as specific components of tumor-permissive ECMs, are interesting candidates for potential exploitation for cancer therapies. Herein, we will briefly revisit the tenascin family with a focus on their structure and patterns of expression in physiological as well as in cancerous conditions. We will further focus on their cancer-related functions providing the basis for their exploitability and value for translational applications and clinical benefit.

## 2 A Brief Encounter With the Tenascins

**Figure 1:**
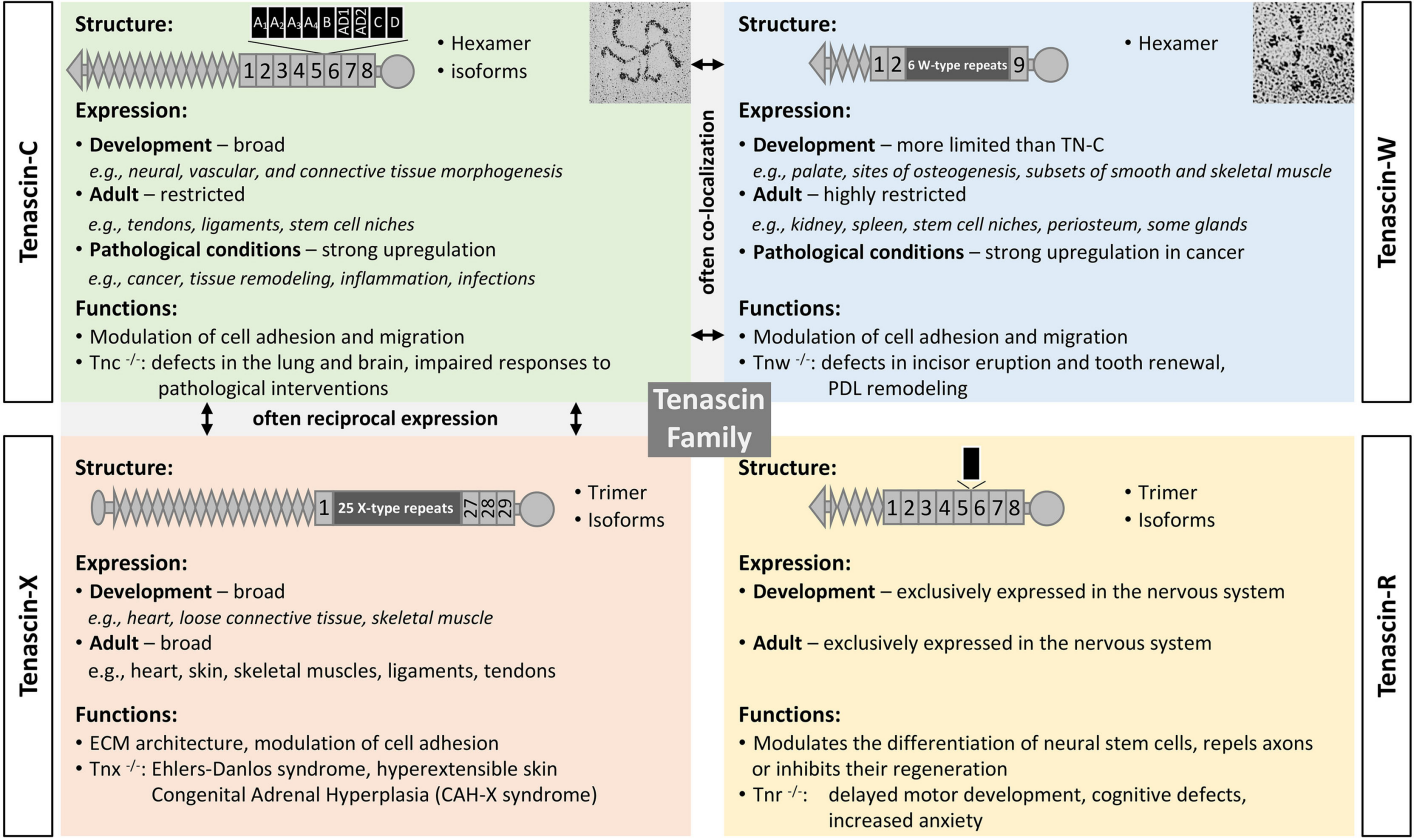
The tenascin family. A brief description of the four tenascin members, including their structure and alternative splicing, major sites of expression, and functions. Original rotary shadowing micrograph for TN-C is from Chiquet-Ehrismann et al. (13) and for TN-W from Scherberich et al. (14) both with permission. The following symbols have been used to describe the structural domains: EGF-like domains (diamonds), FNIII domains (boxes), fibrinogen globe (circle). FNIII repeats shown above the structure are subject to alternative splicing (black boxes). Selected references: for TN-C (15–17), TN-X (18–21), TN-R (22–24), TN-W (25–27), and for tenascins in general (28–30).

Tenascins are a family of modular ECM glycoproteins (**Figure 1**). The family members share both a basic domain architecture and a common evolutionary ancestor. Near their amino-termini most tenascins have a coiled-coil domain that supports trimerization. This is followed by one or more EGF-like domains, numerous fibronectin-type III (FNIII) repeats, and a fibrinogen-like globe (FBG) at their carboxy-termini. The founding member of the tenascin family is TN-C, with the C designating “cytotactin”, which was one of its original monikers. This was followed by descriptions of tenascin-R (TN-R), tenascin-X (TN-X) and TN-W (31, 32). While the roles of the EGF domains remain to be clarified, one or more of a tenascin’s FNIII repeats typically contains integrin-binding motifs (33, 34), and tenascin FBGs bind to and activate toll-like receptor 4 (TLR4) (35). Tenascins are only found in chordates, with all four family members present in bony fishes and tetrapods (36).

After considering each member of the tenascin family, this review emphasizes what we have learned about the two tenascins that are most abundant in the TME: TN-C and TN-W. In humans, TN-C has 14 EGF-like domains and 8 constant FNIII repeats. Between the 5^th^ and 6^th^ constant FNIII repeats alternative splicing can add up to 9 additional FNIII repeats, resulting in hundreds of TN-C variants with significantly different molecular weights (32, 37). The potential to exploit differential expression patterns of TN-C isoforms in normal tissues and in cancer is described below. Human TN-W has just three EGF-like domains and 9 FNIII repeats. There is no evidence of alternative splicing in TN-W (32). In addition to trimerization *via* the coiled-coil domain, the amino-termini of TN-C and TN-W have free cysteine residues that support the formation of hexamers from two trimers (i.e., hexabrachions). As well as being integrin ligands, both TN-C and TN-W can bind to Wnt3a (38).

**Figure 2:**
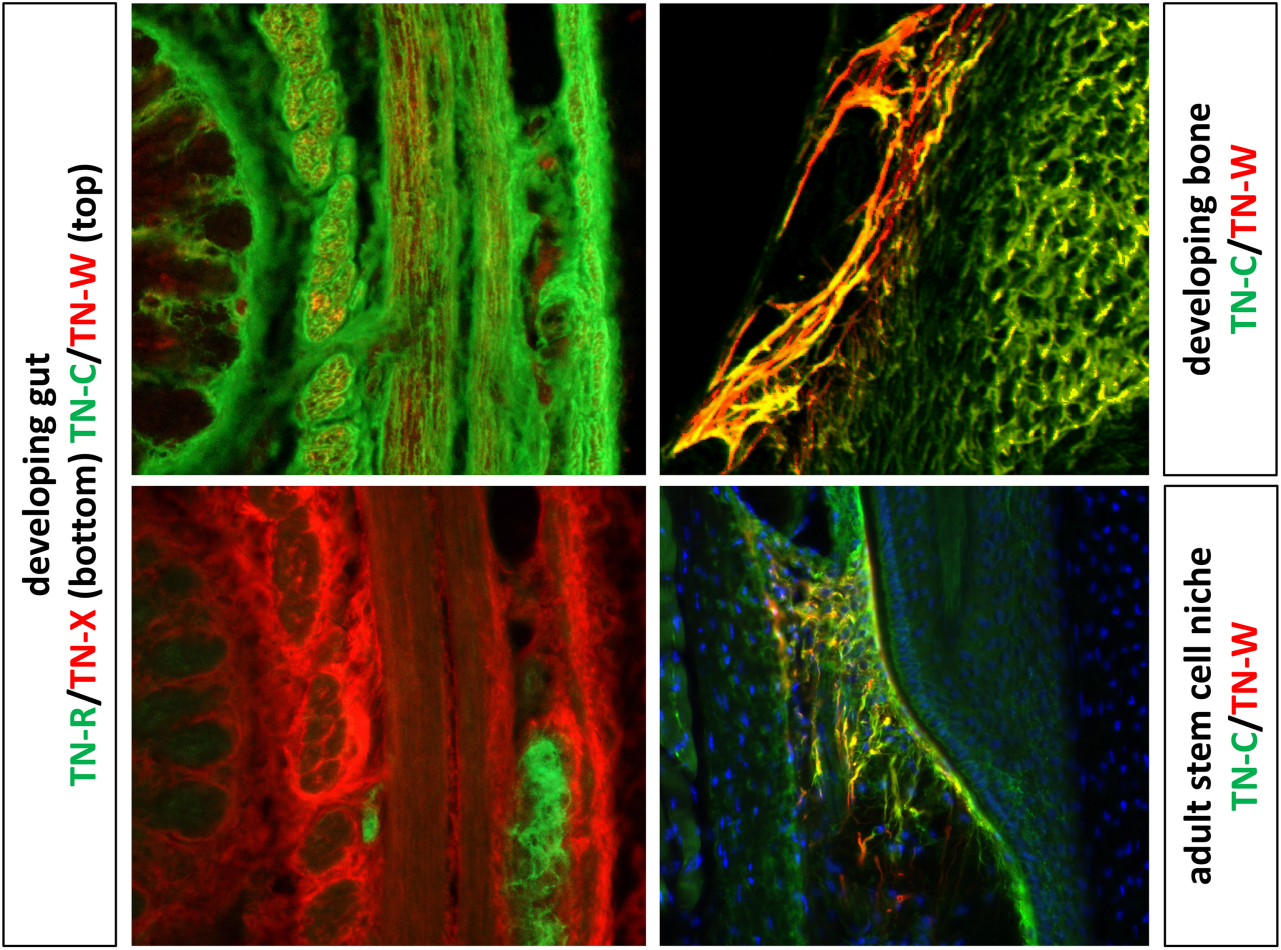
The images to the left show double label immunohistochemistry of the developing gut with antibodies to TN-C, TN-W, TN-R and TN-X. TN-C expression is widespread, while TN-W is found in small patches in a subset of the smooth muscle. TN-R is limited to the autonomic ganglia of the gut, while TN-X is concentrated in the epimysium surrounding bundles of smooth muscle. On the top right is a section of a developing ulna immunostained with antibodies to TN-C and TN-W. Both are strongly expressed in developing bone, with partially overlapping patterns (yellow). On the bottom right is a section though the whisker follicle stem cell niche of an adult mouse. The patterns of TN-C and TN-W expression are more limited in the adult, but they are found in partly overlapping patterns in this stem cell niche. Original images of the developing gut and bone from Meloty-Kapella et al. (40) and adult stem cell niches from Tucker et al. (41) with permission.

TN-C and TN-W are both widely expressed during embryonic development. For example, TN-C is found around migrating neural crest cells, at sites of branching morphogenesis and epithelial-mesenchymal interactions, and throughout the central nervous system in zones where glial precursors are proliferating and migrating. TN-C and TN-W are both expressed at sites of smooth muscle morphogenesis as well as chondrogenesis and osteogenesis, where their expression patterns often partially overlap (32, 37). In the adult, both remain highly expressed in dense connective tissues (28) and in certain stem cell niches (39), but they are largely absent from most other tissues (**Figure 2**).

## 3 Expression of the Tenascin Members in Cancer

The TME plays an active role in cancer progression. Therefore, full awareness of the composition and expression patterns of tumor-specific ECM, compared to the matrix of corresponding healthy tissues, is of high clinical importance. Tumor-specific ECM molecules might be harnessed and targeted in combination with more classical anti-cancer treatment approaches for the benefit of patients. Almost 40 years ago, TN-C was identified as an ECM protein enriched in the stroma of glioma (42). As other members of the tenascin family were identified, each was studied in turn to see if it was also expressed in tumor stroma. The next section will focus on the expression patterns of all four members of the tenascin family in solid cancers.

### 3.1 TN-C: “The Founding Member”

As described above, TN-C is transiently expressed in a highly spatio-temporal manner in various tissues during development (**Figure 2**). In the adult, its expression is only preserved in a few tissues that mainly have to withstand tensile stress (e.g., tendons, ligaments) (28). A robust re-expression in solid cancers is one of the most intriguing features of TN-C. One of the earliest studies analyzing tumor-specific TN-C expression was performed in mouse models of mammary breast cancer (43). These data described TN-C as an ECM molecule present in malignant breast cancer, but absent in the normal healthy mammary parenchyma as well as in benign tumors of the breast. This initial discovery fueled subsequent studies analyzing various cancers for the presence of TN-C, which resulted in an extremely rich literature on its expression in human tumors. TN-C has been identified in the tumor stroma of basically all cancer types analyzed, including cancers derived from breast, brain, uterus, ovary, prostate, pancreas, colorectal, kidney, biliary tract, stomach, gastric, osteosarcoma, oral cavity, urinary tract, skin tissue and many more [for reviews see (44–47)]. This abundant tumor-specific TN-C expression is compelling as TN-C is only mildly expressed in normal adult tissues, although most of them are not completely deprived of it. In certain cancers, such as renal cell carcinoma, brain and lung cancer, TN-C is expressed adjacent to CD31-positive vascular endothelial cells (48). TN-C is not only abundantly expressed in the tissues of cancer patients, but also detectable in their sera. Several studies reported increased TN-C levels in serum of cancer patients compared to healthy controls in non-small cell lung cancer, squamous cell carcinoma of the head and neck, colorectal cancer, breast cancer, and ovarian cancer (49–52).

The *TNC* gene is subject to extensive alternative splicing within its FNIII repeats (**Figure 1**). Hence, many different TN-C isoforms can be produced, with the larger isoforms with additional FNIII repeats typically present during development and in tumors. Indeed, the TN-C isoform spectrum present in the ECM surrounding cancer cells significantly differs from the one in normal ECM (53–56). Consequently, not only general detection of TN-C in tumor stroma is important, but also knowledge about the precise expression of tumor-specific TN-C isoforms is relevant for translational use, as different isoforms in the stroma might affect cancer cell behavior in a distinct way (57). While alternative splicing adds another level of complexity to the multifunctional activities of TN-C, tumor-specific TN-C isoforms also represent promising exploitable targets for anti-cancer therapies (58, 59).

The observation of a tight regulation of TN-C expression during development, in the adult, and in cancers prompted numerous studies trying to elucidate the regulatory mechanisms for this multifunctional ECM molecule. Many factors have been identified, both *in vivo* and *in vitro*, that lead to induction of TN-C. Inducing stimuli include patterning genes during development such as paired-related homeobox 1, as well as growth factors (e.g., PDGF, TGFβ1, FGF2), inflammatory cytokines (e.g., IL1α, IL1β), biophysical properties of the microenvironment (e.g., hypoxia, ECM stiffness), mechanical stress, activated signaling pathways, and micro RNAs (miRNAs). In contrast, glucocorticoids are known to repress TN-C, though the exact molecular mechanism underlying this repression is not clear (60, 61). While some of these regulatory mechanisms might also be relevant for TN-C expression in tumors (e.g., TGFβ1 in breast cancer), the transcription factor SOX4 has been shown to induce TN-C in many malignancies (62). In addition, a crosstalk between TN-C and Notch signaling has also been described in glioma (63). The proximal *TNC* promoter contains a crucial RBPJκ binding element and in glioblastoma, TN-C was shown to be trans-activated by Notch2 signaling in an RBPJκ-dependent manner. How alternative splicing of the *TNC* is gene is regulated is not well understood. Apart from some specific splice factors (e.g., Sam68), certain growth factors/cytokines as well as biophysical properties of the microenvironment might also be involved in the splicing regulation of *TNC* (64–67). Clearly, regulation of *TNC* is a very complex process that reflects its highly specific expression pattern both in physiological conditions as well as in cancers.

*TNC* mRNA has been detected both in stromal as well as in cancer cells. Epithelial-mesenchymal interactions between cancer cells and stromal cells results in the differentiation of quiescent fibroblasts into CAFs, which show a unique gene expression profile. TN-C is one of the specific proteins that is secreted and incorporated into a tumor-permissive ECM by CAFs (8). Furthermore, hyperplastic endothelial cells are also a source for TN-C, which showed strong *TNC* expression in astrocytoma but not in vessels of normal brains (68). Similarly, *TNC* was also identified as a gene highly enriched in endothelial cells isolated from a panel of malignant compared to normal tissues (69). However, TN-C expression has also been found in many cancer cells, including cancers of the breast, brain, skin, colon, pharynx, and larynx as well as in oral squamous cell carcinoma (46). The strongest TN-C positivity is often observed in the cancer cells at the periphery of malignant nests suggesting a role for this tenascin in driving invasion and migration (70). The importance of epithelial-mesenchymal interaction for TN-C expression is illustrated by the fact that in co-culture experiments cancer cells are able to induce TN-C expression in otherwise TN-C-negative fibroblasts (71), while TN-C-negative cancer cells start to express this tenascin when co-cultured with embryonic mesenchyme (72).

### 3.2 TN-R: “The Brain-Specific Member”

TN-R shows a very tissue-specific expression pattern during development and in the adult as it is only detectable in the nervous system (**Figure 2**) (22, 73). Relatively few studies focused on the expression and function of TN-R in cancers, and its expression was only revealed in brain cancer. As often is the case with a low number of studies and lack of functional analyses, the expression as well as the role of TN-R in brain cancer remains controversial. Although TN-R was found to be expressed in fetal cerebellum, TN-R was not detectable in the most common malignant brain tumor in children, medulloblastoma, as assessed by a cDNA microarray as well as immunohistochemistry (74). In contrast, analysis by high resolution northern blot displayed *TNR* in both astrocytoma and meningioma (75). Ayachi et al. show by quantitative real-time PCR (qPCR) and *in situ* hybridization (ISH) that *TNR* was over-expressed in pilocytic astrocytoma and ganglioglioma, two non-invasive tumors, while in low-grade infiltrative glioma and glioblastoma, *TNR* expression was moderate and negative, respectively (76). These results suggest that TN-R could act as a suppressor of glioma invasion. But clearly, more studies are required to allow a conclusive verdict to be given about the role of TN-R in brain and in other cancers.

Regulation of *TNR* remains not well-understood at the moment. Some growth factors and cytokines, including PDGF and NGF, have been shown to induce TN-R *in vitro*. TN-R is mainly produced by oligodendrocytes. Co-culturing these cells with either astrocytes, neurons, or conditioned medium from activated microglia modulates TN-R expression (60). Whether any of these or related mechanisms play a role in aberrant TN-R expression in cancer is not known.

### 3.3 TN-X – “The Controversial Member”

TN-X is found in epimysium and loose connective tissues (**Figure 2**) (32). As with TN-R, only a few studies have addressed TN-X expression in tumor-specific ECMs, and the results of these studies have proven to be controversial. TN-X levels were found to be elevated in the tumor stroma of malignant mesothelioma, ovarian cancer, and low-grade astrocytoma. In the latter, TN-X was expressed in the adventita and the perivascular niche (77–80). In mesothelioma, high TN-X expression allowed the differential diagnosis between malignant mesothelioma and lung adenocarcinoma (78) as well as between malignant mesothelioma and ovarian carcinoma/peritoneal serous carcinoma (77). TN-X is also expressed by Hodgkin lymphoma-derived Reed Sternberg cells (81). Additionally, elevated TN-X concentrations were detectable in serum of breast cancer and ovarian cancer patients compared to control serum (79). Contradictory results were observed in high-grade astrocytoma, malignant nerve sheath tumors, melanoma, and cancers of the uterus (leiomyoma) (80, 82–84). In these malignancies, TN-X was markedly reduced compared to their corresponding normal tissues or to their benign forms. The most elaborate study on TN-X expression in cancer, which used *in silico* analyses of expression data as well as immunohistochemical screening of tissue microarrays, was recently published (85). In this study, TN-X was found to be downregulated in the six cancers with highest incidence and mortality in the world (lung, breast, colon, prostate, stomach, and liver) as well as in all other cancers tested. Notably, diminished TN-X levels were found in the cancers reported to have upregulated TN-X expression by others (see above). Only in brain cancers (astrocytoma, glioblastoma multiforme and oligodendroglioma) was TN-X found to be enriched compared to healthy tissue. For lung adenocarcinoma and breast cancer, clinical data matching the *in silico* datasets were also available, which allowed to establish a negative correlation between TN-X expression and tumor stage as well as patient survival. These data suggest that TN-X levels are reduced in most cancers and that TN-X might represent a promising prognostic biomarker (85). Tumor-specific TN-X downregulation was also shown in another study examining The Cancer Genome Atlas for ECM molecule dysregulation in a large panel of malignancies (86). While 58 of the 249 ECM molecules analyzed were dysregulated in cancers, *TNXB* was found to be the most significantly downregulated gene overall. However, all these data have to be interpreted carefully since (i) TN-X RNA and protein levels did not always correlate; (ii), TN-X downregulation was not observed in every clinical specimen analyzed from the same cancer type; (iii) TN-X was still readily detectable in cancer tissues. However, these observations definitely warrant additional studies looking at TN-X expression and function in cancers.

Very limited data are available regarding *TNXB* gene regulation. So far, there is no evidence that *TNXB* responds to any growth factors or cytokines, which is in contrast to the other tenascin members. This is somewhat surprising since several putative binding sites for Sp1/Sp3 transcription factors were identified close to the transcription start site and found to be functional and required for driving *TNXB* expression. However, the upstream signaling pathways remain elusive. As with other tenascin family members, it is known that glucocorticoids have a negative effect on *TNXB* levels. However, details remain unresolved (60).

### 3.4 TN-W: “The Most Tumor-Specific Member”

TN-W is the newest member of the tenascin family, which was often found co-expressed or at least in the vicinity of TN-C during development (**Figure 2**) (25, 26). Since TN-C has been known for its high expression in the tumor stroma of breast cancer, initial studies on TN-W in tumors focused on this tissue as well. Using different breast cancer mouse models, TN-W was found to be enriched in the tumor stroma surrounding breast cancer cells. However, tumor stroma was only positive for TN-W in the models with a high likelihood to metastasize and not in the non-metastatic ones (87). Encouraged by these findings in mice, the human orthologue was cloned, expressed, and antibodies raised against it. The first tissues analyzed resulted in intriguing observations. In healthy normal breast and colorectal tissues, TN-W was not detectable at all, while their corresponding cancerous tissues displayed a robust and highly significant *de novo* expression of TN-W (52, 88). Screening of additional human tumor types confirmed these initial observations: TN-W was strongly enriched in the stroma of brain (glioblastoma, oligodendroglioma, astrocytoma), prostate, kidney (clear cell carcinoma, papillary carcinoma, chromophobe renal carcinoma, and oncocytoma), ovarian, prostate, pancreas, biliary tract (liver carcinoma, gallbladder carcinoma), and lung cancers as well as in melanoma (48, 89, 90). Most significantly, TN-W was not detectable in the corresponding healthy tissues. However, TN-W expression in tumors was heterogenous, varying from low to very high levels, but was significantly increased compared to the normal tissues in most of the cases tested. For some metastatic melanoma patients, lymph node metastases as well as metastases from diverse locations (spleen, lung and skin) were found to be TN-W positive (87.5% of lymph node metastases, and 85% of other metastases) (48). Similar to TN-C, TN-W was often found to be expressed in close proximity to the vasculature as assessed by co-staining of TN-W with endothelial cell markers CD31/Pecam-1, von-Willebrand-factor, and Desmin in at least kidney, lung, breast, colon, ovary, and brain cancers (48, 90). Elevated concentrations of TN-W could also be measured in the serum of breast and colon cancer patients compared to healthy controls using a sensitive TN-W sandwich ELISA (52).

Although the list of solid cancers with prominent upregulation of TN-W is constantly growing, some very elementary questions about TN-W in tumor stroma remain. For instance, what is the cellular source of its expression? It is believed that TN-W is mainly expressed by stromal cells mediated by epithelial-mesenchymal interactions. This statement is supported by immunohistochemical analyses of TN-W that never revealed any TN-W expression in cancer cells as well as by *in vitro* experiments unsuccessfully trying to detect endogenous TN-W in numerous cancer cell lines of various origin (26). The concept of stromal cell origin of TN-W was reinforced by observations that bone marrow-derived stromal cells exclusively expressed TN-W when co-cultured with malignant cells in a xenograft model of breast cancer cell displaying metastatic propensity toward bone (91). However, a recent study screening for *TNW* transcripts in 20 biliary tract cancer cell lines is challenging the idea about pure stromal cell origin of TN-W (89). While most of the cells displayed very modest or negligible *TNW* levels, Huh-28 cells, an intrahepatic cholangiocarcinoma cell line, showed high levels of *TNW*. These results were further confirmed by immunoblotting and immunocytochemistry. Notably, when cultured alone, TN-W in Huh-28 cells was mainly observed to be intracellular without being organized and incorporated into the ECM. However, when co-cultured with bone marrow-derived stromal cells, Huh28-derived TN-W organized into ECM fibrils surrounding nests of tumor cells, a situation that closely mimics the situation of tumors *in vivo* (89). Therefore, there is strong evidence that proper epithelial-mesenchymal crosstalk is required for formation of TN-W-positive fibrils, but also that cancer cells themselves might represent a cellular source for TN-W, which is similar to the situation for TN-C. Additional experiments, such as ISH on tumor samples, are required to fully elucidate the cell types responsible for TN-W expression and to know whether the cellular source might differ among distinct tumors.

Another open question about TN-W expression in tumor stroma concerns the regulatory mechanisms of its *de novo* expression. Most of the available knowledge on this topic has been gained in *in vitro* studies of mouse and chicken cells. Growth factors, such as BMP2, BMP7, and TNFα induce TN-W in mouse embryonic fibroblasts, mouse cranial fibroblasts, mouse C2C12 myoblasts, HC11 normal mouse mammary gland epithelial cells, mouse bone marrow-derived Kusa-A1 cells as well as in primary chicken osteoblasts (14, 92, 93). In addition, Wnt5a seems also indirectly involved in triggering TN-W expression through p38/MAPK signaling (94). However, the molecule that acts as the upstream inducer has not been identified. In contrast, none of these factors have been shown to be able to activate *TNW* in human cells. Today, we only know that TGFβ1 signaling has an inducing effect on TN-W. Breast cancer cells secrete TGFβ1 into the tumor stroma, where the growth factor is sensed by bone marrow-derived stromal cells, which leads to a Smad4-dependent induction of *TNW* in the stromal cells (91). Negative impact on *TNW* transcripts has been attributed to glucocorticoids, which is similar to their effect on *TNC* and *TNXB* (60). More studies and research are required to better understand cancer-specific expression of TN-W.

There is not much evidence available for the presence of specific TN-W isoforms or TN-W modifications. The only observation in this regard was gained in the Huh-28 cells, which showed a TN-W protein with a higher molecular weight than expected (89). Whether this result hints at a tumor-specific isoform, modification, or multimerization of TN-W in this cell line and whether it has any relevance *in vivo* remains to be seen.

## 4 Potential Cancer-Promoting Activities of Tenascins

**Figure 3:**
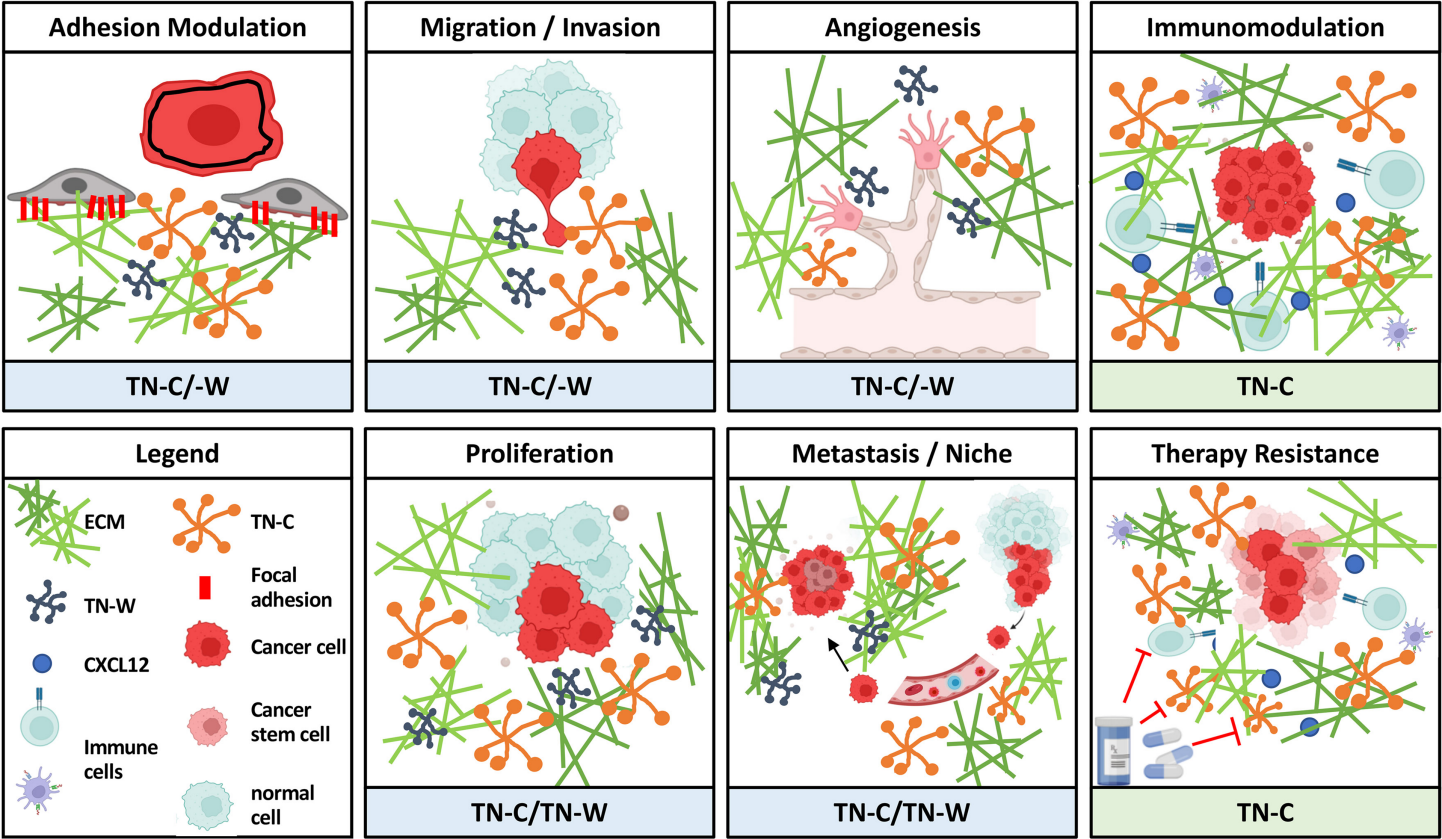
Overview of oncogenic activities of TN-C and TN-W. As matricellular proteins, TN-C and TN-W can influence various cellular processes involved in tumor progression by modulating the interplay between stromal and epithelial cells or other cells. Schemes were created using www.biorender.com.

As mentioned before, tenascins are multifunctional matricellular proteins with a highly specific pattern of expression during development and in cancer. Although each family member might be involved in cancers to some extent (see above), currently there is only robust and consistent data about expression and function in tumors for two of the family members: TN-C and TN-W. Therefore, we put the focus in the following chapters on TN-C and TN-W and try to emphasize their role in regulating various cellular processes required for tumor progression (**Figure 3**).

### 4.1 Modulation of Cell Adhesion

The potential to modulate cell adhesion is probably one of the most described and studied characteristics of TN-C. TN-C is an anti-adhesive molecule, which, as substratum, does not promote attachment and spreading of either epithelial cells (e.g., breast cancer cells) or fibroblasts (95). The most prominent feature of TN-C is its inhibitory effect on fibronectin (FN)-mediated cell adhesion (13). Attachment and spreading on FN requires cells to engage synergistically both the heparan sulfate proteoglycan receptor syndecan-4 and integrin α5β1. However, TN-C can bind to FN at a heparin binding motif located around the 13^th^ FNIII repeat, which leads to a competitive hindrance of syndecan-4 binding to FN. Consequently, the presence of TN-C (such as in tumors) inhibits cell spreading and increases cell proliferation (96). Similar, but less consistent and less understood properties, have been found for TN-W in regulating cell adhesion. Initially, a TN-W substratum was found to be anti-adhesive for human breast cancer cells, while it promoted human fibroblast attachment, but not proper spreading. However, for both cell types, the presence of TN-W did not interfere with FN-mediated attachment (88). Different results were observed in primary chicken osteoblasts, mouse C2C12 myoblasts, embryonic fibroblasts, and whisker follicle stem cells, for which TN-W was able to interfere with FN-mediated cell spreading when soluble or used as substratum (41, 97). These results suggest that the activity of TN-W to modulate cell adhesion and spreading is cell-type specific. For cancer cells, TN-W seems to be anti-adhesive and not able to interfere with FN-mediated cell attachment, while for normal cells, TN-W shares the anti-adhesive properties of TN-C towards FN-mediated cell spreading. However, the mechanistical details underlying the latter observation remain unknown, as direct TN-W/FN interactions have yet to be reported.

### 4.2 Cell Proliferation

*In vitro*, cancer cell lines can be stimulated to become hyperproliferative by the addition of recombinant TN-C (45). This is most likely the consequence of TN-C impairing cell adhesion to FN by blocking the binding site of syndecan-4 (96). As a result, tumor cell proliferation was enhanced in the presence of TN-C. Gene expression profiling revealed the mechanistical details for this effect: TN-C disrupts the actin cytoskeleton by downregulation of tropomyosin 1, while it was able to de-repress and activate Wnt and MAPK signaling, respectively (98). Besides having a pro-proliferative effect on cancer cells, TN-C is also able to stimulate proliferation of endothelial and smooth muscle cells (45). The mitogenic activity of TN-C is supported *in vivo* by immunohistochemistry showing TN-C co-expression with the proliferation marker Ki-67/Mib-1 in many invasive carcinomas (99), and the observation that TN-C is associated with proliferative epithelial cells in regenerating epithelia (100). A study by Swindle et al. also revelaed that select EGF-like repeats of TN-C might promote proliferation in an EGFR-dependent way by activating downstream MAPK signaling (101). In contrast, there are not many studies available on the effect of TN-W on cell proliferation. While an anti-proliferative activity was reported for TN-W in the murine osteoblastic cells MC3T3 (92), treatment of MDA-MB231-1833 breast cancer cells with different concentrations of recombinant TN-W resulted in a significant increase of BrdU incorporation, suggesting that TN-W is able to stimulate cancer cell proliferation (91).

### 4.3 Cell Migration/Invasion

In clinical specimens, TN-C is often expressed in the intratumoral stroma but also at the invasive front of the tumor nests (46). TN-C expression at the invasive front is correlated with aggressive tumor behavior and poor prognosis and is suggestive of a role for TN-C in promoting migration and invasion of cancer cells (45, 46). These observations could be recapitulated *in vitro* by showing increased motility of various cell types, including cancer cells, fibroblasts, endothelia cells, and smooth muscle cells, in the presence of soluble TN-C or TN-C as substratum (102–104). Again, the TN-C-mediated cell adhesion modulating effect might explain the mechanisms underlying the pro-migratory outcome. In the presence of TN-C, cancer cells cannot properly spread, favoring an intermediate state of adhesions with suppressed Rho activation and actin-rich filipodia, which enhances their motile behavior (105). In contrast, lack of TN-C allows cell spreading and the formation of stable focal adhesions with stress fibers. Similar pro-migratory properties can be attributed to TN-W, although the molecular mechanisms have not been elucidated yet. First evidence for this finding has been gained in the highly metastatic murine mammary cancer cell line 4T1, which showed a faster migratory behavior across a transwell filter coated with TN-W (87). A follow-up study using human T47D breast cancer cells lines confirmed this result and showed that treatment of the cells with soluble TN-W increased their migratory phenotype (88). Accordingly, the migration of chicken osteoblasts across filters coated with TN-W was stimulated and TN-W added to the medium increased osteoblast migration when cultured on FN-coated filters when compared to controls (106).

Another prominent feature that is linked to altered motile behavior is the process of epithelial-mesenchymal-transition (EMT). While EMT is crucial during development, cancers often use this process for invasion and their dissemination into distant organs (107). TN-C has been found to be able to promote EMT. In MCF7 breast cancer cells, TN-C induced EMT-like morphological changes, which, on a molecular level, included the delocalization of E-cadherin and β-catenin correlating with tyrosine-protein kinase SRC activation and the SRC-mediated phosphrorylation of FAK (108). Subsequently, these changes were accompanied by cell detachment from the substratum and an enhanced migratory cell phenotype. In clinical breast cancer specimens, a frequent upregulation and co-localization of TN-C with the mesenchymal marker vimentin was observed at the invasive fronts of cancers in those cells having a more scattered and mesenchymal-like morphology. Such a cancer fingerprint could be correlated to increased malignancy in breast cancer (109). These data suggest an active role for TN-C in promoting EMT-like features. In regard to this, a recent *in vitro* study identified the highly conserved C-terminal FBG of TN-C and TN-W as activators of latent TGFβ (110). Subsequently, the mature TGFβ is presented to cells, which elicits an intracellular smad-dependent signaling cascade. Since TGFβ is a potent inducer of EMT (111), these data indicate that the FBGs of tenascins might be able to promote a TGFβ-mediated EMT. Whether this process plays an active role in TN-C- and/or TN-W-rich tumor stroma remains to be elucidated. However, EMT-promotion by tenascins represent intriguing options for them to drive cancer progression.

### 4.4 Metastasis and the “Cancer Stem Cell” Niche

Metastasis is a complex multistep process by which cancer cells gain the power to escape from the primary tumor mass, disseminate into the vasculature and throughout the body, and finally home and colonize distant organ sites to form secondary cancers (112). On this journey, cancer cells must overcome several hurdles and challenges. Successfully disseminated cancer cells often are able to produce a specific microenvironment, a so-called niche, that protects them and enables them longer survival in a hostile environment during their metastatic journey (70, 113). The invasive front of tumors has been suggested to be rich in cancer-initiating cells (“cancer stem cells”) and the same region is known to express high levels of TN-C, indicating that TN-C might be a component of the metastatic niche (70). Disseminating breast cancer cells that express their own TN-C have survival advantages early on until the CAFs take over and produce TN-C, creating a niche that supports their fitness by modulating stemness-like signaling pathways such as Wnt and Notch (70, 114). A more recent study promotes TN-C as a specific and highly-expressed molecule of the lymph node pre-metastatic niche in muscle invasive bladder cancer (115). In this niche, TN-C induction by CAFs is believed to be triggered by cytokines present within tumor cell-derived extracellular vesicles (EV). Hence, infiltrating tumor cells secrete EVs with signaling cues that prepare a TN-C-rich niche, which provides survival advantages (115). Similar findings have recently emerged for TN-W (91). Breast cancer cells on their way to metastasize to bone are surrounded by a specific niche, established by epithelial-mesenchymal interactions. Cancer cell-derived TGFβ1 induces *de novo* expression of TN-W in the stromal cells, providing an important component for the metastatic niche. Accordingly, aberrant presence of TN-W in the metastatic niche, shaped and tailored by the cancer cells, provides the stimuli for increased cancer cell proliferation and migration, thereby increasing the risk for bone metastases (91). *In vitro* studies in biliary tract cancers revealed that TN-W fibrils are only formed in the presence of stromal cells (89). This observation might reflect such a metastatic niche as well. In there, TN-W can carry out its potential oncogenic activities leading to the successful dissemination of the cancer cells.

TN-C is also believed to influence metastasis by its very specific organization in the tumor matrix networks. Together with additional ECM proteins, TN-C remodels the ECM to form channel-forming, migratory tracks, which provides signaling cues for the survival of cancer cells and supports dissemination of cancer cells during the metastatic process (116, 117). Notably, periostin (POSTN), another matricellular ECM molecule associated with the metastatic niche (118), interacts with TN-C and is responsible for TN-C incorporation into this specialized ECM organizational units. While doing so, POSTN might enhance collagen cross-linking, which leads to the formation of migratory tracks during metastasis (119). Functionally, both POSTN and TN-C are known to be able to activate Wnt signaling in cancer cells by recruiting Wnt ligands and the downregulation of the Wnt signaling pathway inhibitor DKK1, respectively (98, 118). The education of stromal cells to produce POSTN and TN-C by infiltrating cancer cells represents a crucial step during the metastatic process, which might be exploitable for anti-cancer therapies.

### 4.5 Angiogenesis

TN-C as well as TN-W are found associated with endothelial cells in various cancers suggesting that both tenascins might play a role in promoting tumor angiogenesis. *In vitro*, both tenascins trigger an elongated morphology and motility of human umbilical vein endothelial cells (HUVEC), properties related to angiogenic endothelial cells (90). Presence of either TN-W or TN-C in a collagen gel induces endothelial cell sprouts in gel-embedded HUVEC spheroids compared to collagen alone (90). Similarly, injection of malignant melanoma cells into TN-C-deficient mice revealed significantly less vascularization within the tumor mass (120). All these data point towards an active role for TN-C and TN-W in promoting angiogenesis in cancers. Indeed, TN-C in the TME contributes to the angiogenic switch in neuroendocrine tumors (121) and belongs to the AngioMatrix signature (a panel of 110 ECM genes) that correlates with poor prognosis in glioma patients (122). In addition, a higher vessel density, but less functional vessels, are also observations attributed to high TN-C levels in tumors (121). How TN-W triggers angiogenesis mechanistically has yet to be addressed. For TN-C, it is known that its presence in the mesenchyme results in increased levels of the pro-angiogenic factor VEGF in A375 melanoma cell transplant experiments (120) and TN-C is able to repress Wnt signaling by DKK1 in tumor and endothelial cells creating a proangiogenic TME (121). Increased complexity was revealed by a more recent study showing that TN-C can regulate both pro- and anti-angiogenic factors (123). Direct contact of TN-C with endothelial cells inhibits YAP signaling by disrupting actin polymerization. Since YAP signaling is required for the induction of pro-angiogenic genes (e.g., *CTGF* and *CYR61*), endothelial cell survival, proliferation, and vessel formation is impaired. In contrast, brain cancer cells exposed to TN-C respond by secretion of pro-angiogenic signals promoting tubulogenesis of endothelial cells. Hence, TN-C can play multiple and opposing roles during tumor angiogenesis depending on the cells with which it interacts.

### 4.6 Immunomodulation

Although it has been known that TN-C is able to shape innate and adaptive immunity (124, 125), its impact on tumor immunity is not fully elucidated yet. Studies have revealed that the C-terminal FBG of TN-C as well as of TN-W is able to activate TLR4 on either macrophages or fibroblasts (35, 126). TLR4 is a key component of the innate immune system and when activated stimulates the secretion of pro-inflammatory cytokines such as IL6, IL8, or TNFα (127). *In vivo* relevance for TLR4 activation by the FBG of TN-C was established in an orthotopic grafting model of breast cancer (128). Application of a therapeutic monoclonal antibody raised against the FBG of TN-C blocked TLR4 activation and reduced primary tumor growth and metastasis (128). The same authors reported a dual role for TN-C on tumor immunity depending on its cellular source: while tumor cell-derived TN-C polarized macrophages towards a pathogenic, immune-suppressive phenotype, host stromal cell-derived TN-C promoted immunity by recruiting anti-tumoral macrophages. Additional roles for TN-C in modulating the immune system in cancers are only recently emerging. In a mouse model, prostate cancer-initiating cells were protected from immune surveillance by over-expressing TN-C, which in turn was able to inhibit T-cell proliferation and effector functions (129). Similar results were obtained in a study in brain cancer. There, TN-C is packaged into exosomes derived from brain tumor-initiating cells and released into the microenvironment, where it can suppress systemic T-cell responses through an α5β1 or αvβ6 integrin-dependent inhibition of T-cell activation and proliferation (130). Both studies promote TN-C as a molecule providing protection for cancer-initiating cells from host-derived immune surveillance. Moreover, TN-C was recently shown to contribute to an immune-suppressive microenvironment in oral squamous cell carcinoma by mobilizing dendritic cells in the tumor stroma through binding CCL21 (131). Similarly, TN-C was responsible for the induction of CXCL12, which in turn immobilized CD8^+^ tumor infiltrating T lymphocytes (CD8 TIL) in the matrix. Trapped in the TN-C-rich matrix, CD8 TIL could not combat and kill breast cancer cells, suggesting a role for TN-C in anti-tumor immunity escape (132). The newest discovery that the FBGs of both TN-C and TN-W can activate latent TGFβ renews speculation about a potential involvement of tenascins in tumor immunomodulation (110) since TGFβ is a potent suppressor of immune responses (133). Data on the effect of TN-W in immunomodulation remain absent apart from the potential of its FBG to activate TLR4 and TGFβ. Whether such activities might play a role in tumor progression has to be addressed in the future.

### 4.7 Therapy-Related Activities

Cancer therapy resistance represents a major hurdle and some evidence suggests that TN-C might be involved in this process. In a melanoma model, knockdown of TN-C not only significantly diminished the stem cell-like cells in melanoma spheres, but also lowered their resistance to doxorubicin treatment (134). Therefore, it can be assumed that TN-C is a driver of melanoma progression as it provides protective cues for therapy-resistant melanoma-initiating cells. A tumor-specific large TN-C isoform was shown to confer gemcitabine resistance in pancreatic cancer cells by the canonical phosphatidylinositol 3-kinase/AKT/NF-κB signaling pathway by its interaction with Annexin2 (135). In a study aiming to determine changes of stromal proteins in breast cancer following neoadjuvant chemotherapy, TN-C was identified as a molecule being important for conferring cancer cell resistance to doxorubicin- and docetaxel-based neoadjuvant chemotherapy by activating the integrinβ1/mTOR pathway (136). Moreover, a correlation of high *TNC* expression with tamoxifen resistance was identified in a clinical study analyzing 1286 primary breast tumors by qPCR (137).

Having reviewed the functions of TN-C in tumor immunity, TN-C might also affect the success rate of anti-cancer immunotherapies. Regarding this, a recent study identified a molecular mechanism explaining the observation that triple-negative breast cancers (TNBC) often do not respond to T-cell-mediated immunotherapies (138). In TNBC, defective autophagy-mediated TN-C degradation results in TN-C accumulation, which impairs T-cell-mediated tumor cytotoxicity. This phenotype could be reversed by inhibition of TN-C suggesting an important role for TN-C in autophagy deficiency-mediated immunosuppression. Consequently, blocking TN-C by specific anti-TN-C antibodies could boost the efficiency of immunotherapies in TNBC.

In addition to chemo- and immunotherapies, radiotherapy is a frequent cancer treatment option. Radiotherapy triggers DNA damage leading to the death of rapidly dividing cancer cells. However, tissues adjacent to the tumor and other normal cells are also affected. Ionization can lead to the modulation of the ECM and indeed, TN-C has been found to be induced upon this treatment (47). Moreover, it is known that ionization also stimulates an inflammatory reaction, fibrosis, and hypoxia. These conditions are known to mediate high TN-C expression (28). With all the knowledge we have gained about the tumor-promoting activities of TN-C, radiation, while killing cancer cells, might also create a pro-tumorigenic stroma. Whether or not this radiation-induced TN-C plays a role in potential tumor relapses, or secondary tumor formations after radio-treatment, remains to be investigated. Nothing is known yet about TN-W function in therapy resistance.

## 5 Can We Make Use of the Prominent Expression of TNC and TNW in Tumor Stroma?

Having summarized the expression patterns of TN-C and TN-W in tumors as well as their potential tumor-promoting activities, we plan to shed light on the possibilities they offer to be harnessed for clinical use and for the benefit of cancer patients.

### 5.1 Diagnostic and/or Prognostic Tumor Biomarker

**Figure 4:**
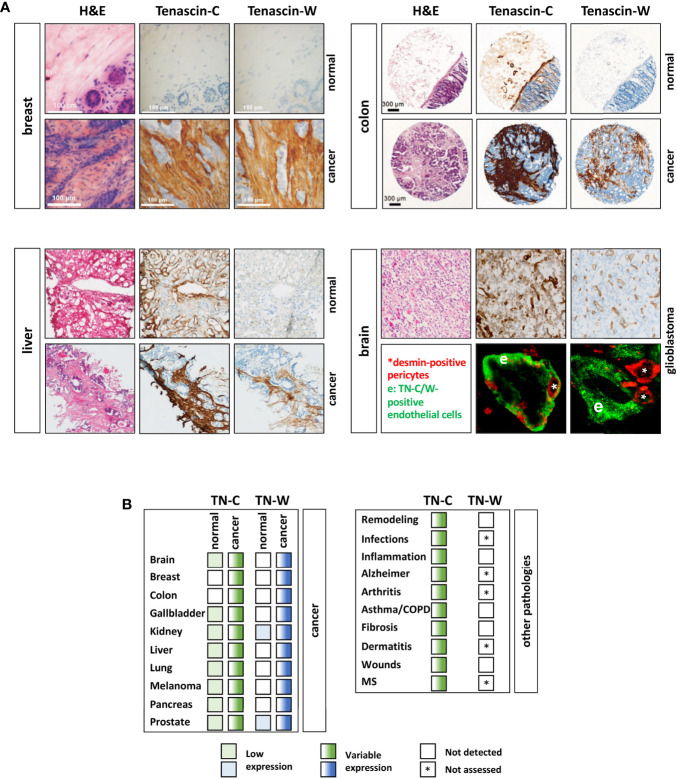
TN-C and TN-W expression in human cancers. **(A)** Examples of immunohistochemical analyses of TN-C and TN-W in human breast, liver, and colorectal cancer as well as in adjacent normal tissues. Note that while TN-W is not expressed in healthy tissues, TN-C is detectable in colon and liver. In glioblastoma TN-C and TN-W show strong expression around vessel-like structures (top). Double immunofluorescence staining of TN-W or TN-C (green) with desmin (red) shows that TN-C encloses all pericytes, while TN-W staining is adjacent to them. Original images of breast tissue from Degen et al. (88), of liver tissue from Hendaoui et al. (89), of colon tissue from Degen et al. (52), and of brain tissue from Martina et al. (90) with permission. **(B)** Table of all cancer/normal tissues that have been analyzed so far for TN-W and TN-C by immunohistochemistry and/or immunoblotting (left). Right table shows non-cancerous conditions with TN-C upregulation and non-detectable (white box) TN-W expression. Fully colored boxes: expressed; gradient-colored boxes: variable expression ranging from low (light) to high (dark). * not assessed yet; MS, multiple sclerosis.

Cancers are most dangerous when they are able to form distant metastases. Unfortunately, some cancers are only detected when such metastases have already formed rendering therapy even more challenging. In order to prevent this, there is an urgent need for diagnostic tumor markers that recognize premalignant lesion with high specificity and sensitivity. The best-case scenario would be that in adult healthy tissues a potential biomarker is not detectable, while its levels are sharply elevated in early premalignant dysplasia. This would allow an early detection of dysplastic and pre-malignant tissues. Both TN-C and TN-W seem to be interesting cancer biomarker candidates as they are often over-expressed in the tumor stroma (**Figure 4A**). However, the two tenascins differ significantly in their expression in healthy and noncancerous tissues. A recent screening of a healthy tissue microarray revealed limited basal TN-W expression. Apart for some expression in spleen, kidney, and some adult stem cell niches (e.g., hair follicles), as has been already observed in the initial mouse studies, TN-W was also weakly present in the female reproductive system and in certain glands (89). In contrast, TN-C is readily detectable in a larger array of healthy tissues (**Figure 4A**). Moreover, it is well established that TN-C upregulation is not restricted to cancers, but that abundant TN-C expression is a prominent feature of many other pathological conditions. These include inflammatory diseases (e.g. arthritis and asthma), infections (e.g. viral infections), and tissue remodeling processes such as wound healing and fibrosis (28). This is in stark contrast to TN-W, for which there is no evidence for *de novo* expression in pathological conditions other than cancers. Even in conditions with strong TN-C expression such as healing wounds and inflammatory bowel diseases, TN-W is not detectable (**Figure 4B**). These observations promote TN-W as a better and more tumor-specific tissue biomarker than TN-C (48). However, we have mentioned that TN-C is subject to alternative splicing generating very tissue- and condition-specific isoforms. Therefore, it might be also worth studying tumor-specific expression of distinct TN-C isoforms. It is known that certain splice variants, especially the variants with additional FNIII domains, are expressed in cancerous conditions. Hence, even if normal healthy tissue is expressing TN-C, detection of tumor-specific TN-C isoforms might still be of clinical value as a diagnostic readout.

TN-C and TN-W can also be detected in elevated concentrations in serum of cancer patients. But similar to the situation in tissues, increased TN-C levels in serum are also found in additional pathologies. Some of the conditions known to have increased serum TN-C levels include spondylitis, sepsis, myocardial defects, traumatic brain injuries, rheumatic diseases, pulmonary hypertension, Alzheimer’s disease, systemic lupus, idiopathic inflammatory myositis, muscular dystrophy, chronic hepatitis C infection, and chronic renal diseases (139–146). Common to most of these conditions is the fact that they involve an inflammatory reaction. Therefore, it is not surprising that TN-C in serum correlates with C- reactive protein, a common inflammation marker, and represents a questionable tumor biomarker (147, 148). As mentioned above, the presence of certain TN-C splice variants in serum may still have some value as a marker of cancer. In contrast, to date elevated TN-W has only been detected in the serum of cancer patients (52).

Numerous studies also assessed the prognostic value of TN-C. Although these results are often ambiguous, there seems to be a clear trend towards a positive correlation between TN-C levels and local tumor recurrence, the metastatic potential, and poor prognosis in many aggressive human cancers. This correlation was observed in clinical specimens of brain, breast, colon, head and neck, and lung cancer (45). Why this observation is not valid for other cancers is not understood, but it might reflect the multifunctional roles of TN-C and tumor heterogeneity. There are also two studies that report a correlation of serum TN-C with prognostic factors of cancers: In non-small cell lung cancer patients TN-C serum levels correlated with larger tumor size, lymph node metastases and patient’s overall survival (51), while serum TN-C levels in grade 3 breast cancer were significantly higher than in grade 1-2 tumors (50). Moreover, TN-C is known to be a substrate for various matrix metalloproteinases and serine proteases, which can lead to TN-C fragmentation (149). Significantly more small TN-C fragments were found in lung cancer patients who had a higher risk to develop lymph node as well as poor overall survival suggesting that TN-C degradation might be a marker for the metastatic potential of lung tumors (150, 151). Unfortunately, little has been done to date to study the prognostic value of TN-W. Initially, TN-W was found to be mainly enriched in mouse models of mammary cancer that metastasize (87). However, such a correlation could not be confirmed in human breast cancer where TN-W was more strongly expressed in low-grade tumors than in high-grade tumors (88). In lung cancer, TN-W showed a tendency towards higher expression in high-grade compared to low-grade cancers. Similarly, the more severe clear cell carcinoma expressed more TN-W (and TN-C) than the less severe oncocytoma (48). In colorectal carcinoma patients with tumor recurrence, serum TN-W levels were higher than in those without recurrence (52). Although these data suggest some prognostic value of TN-W in certain malignancies, the number of cases analyzed for TN-W are too small to draw any definite conclusions yet.

### 5.2 Modulating Tumor-Specific Tenascin Expression

TN-C and TN-W are enriched in most solid human tumors. Both molecules also share a broad spectrum of tumor-promoting activities (**Figure 3**). Reversing their high expression in tumors could be a promising concept to normalize the TME thereby impairing its effect on promoting tumor progression. This approach should be attempted in combination with more classical, cancer cell-targeting therapies with the ultimate goal to limit the metastasis of, and to kill, cancer cells. To target tumor-expression of tenascins and prevent TN-C and TN-W from joining the TME, there are broadly two options: pharmacological inhibition and gene-based approaches.

#### 5.2.1 Pharmacological and Natural Inhibitors

A simple concept for reducing the expression levels of tenascins includes the inhibition of the signaling pathways required for to their activation. However, this approach requires full knowledge about how tenascins are regulated in tumors. While for TN-C several pathways have been reported to lead to its induction (e.g. TGFβ signaling), knowledge of TN-W regulation in tumors remains sparse. In addition, inhibiting such important and canonical pathways will likely have unwanted side effects and therefore, does not represent a feasible approach to target tenascins in tumor stroma.

The use of miRNAs might represent an alternative approach to target tenascins. miRNAs are regulators of post-transcriptional gene regulation, which are also known to play a critical role in different cancer types as their dysregulation has been shown to affect the hallmarks of cancer (152). Therefore, tenascin-specific miRNA could be utilized in combination with additional treatment strategies to increase treatment success against cancer. A major challenge for this approach is the efficient delivery of these agents to target cells, which might require antibodies or peptides. Several miRNAs have been identified to suppress TN-C levels in cancers: miRNA-198 in colorectal cancer (153), miRNA-335 in breast cancer (154), miRNA-150 in head and neck squamous cell carcinoma (155), and miRNA-218 in glioma (156). In brain cancer, miRNA-107 indirectly diminishes TN-C levels by targeting Notch2, a direct inducer of TN-C (157). So far, no data exist about a possible regulation of TN-W by miRNAs.

Glucocorticoids are known repressors of both TN-C and TN-W (60). Therefore, the use of corticoids, which dampen tenascin expression, might represent a promising addition to the treatment cocktail of specific cancer types. Such an approach is applied in the treatment of castration-resistant prostate cancer patients, who receive dexamethasone as monotherapy or as part of their chemotherapeutic cocktail (158, 159). However, the effects of a widespread use of dexamethasone either to attenuate the side effects of chemotherapy or to combat malignancies might be double-edged as it has been shown that the glucocorticoid can enhance lung metastasis of breast cancer cells (160).

Recently, an approach to reduce TN-C in the tumor stroma of pancreatic cancer involved interfering with the aberrant cancer cell-mesenchymal crosstalk, which is required for stellate cell activation (161). These activated stellate cells are responsible for TN-C secretion into the stroma, in which TN-C can activate oncogenic signaling cascades (Wnt/β-catenin *via* inhibition of DKK1 and YAP/TAZ signaling) leading to tumor progression. The metastasis suppressor, N-myc downstream-regulated gene-1 (NDRG1) is able to dampen tumorigenesis by inhibiting these two pathways as well as by decreasing TGFβ production by cancer cells. The latter factor is required for stellate cell activation, which enables TN-C synthesis. Consequently, upregulation of NDRG1 might have the power to normalize the TME by impairing stellate cell activation thereby preventing induction of TN-C. Interestingly, the anti-cancer compounds thiosemicarbazone potently upregulate NDRG1, which both prevents aberrant epithelial-stellate cell crosstalk and synthesis of TN-C.

#### 5.2.2 Gene-Based Approaches

Another strategy for anti-cancer treatment includes the use of RNA interference (RNAi) technologies. With RNAi, it is possible to specifically target a gene that is known to play a key role in tumor progression. As such it is not surprising that several attempts used RNAi to target TN-C in cancers (**Table 1**). Double-stranded RNAs specific for the TN-C sequence (ATN-RNA) were used to inhibit its expression in glioma, which are rich in TN-C. ATN-RNA was injected into the brain of 11 patients after initial tumor surgery, and treatment specificity and effects were assessed (162). Thereafter, the study was extended to 46 patients with grade II, III, and IV glioma (163, 164). Both the well-being as well as progression-free and overall survival of the patients were significantly increased in the ATN-RNA treated patients compared to the ones only obtaining brachytherapy. Although promising, this treatment only provided small advantages in survival prolongation. Whether the foreign dsRNA elicited an immune response affecting the patient’s outcome was not assessed. A recent study used the same approach *in vitro* to show proof of concept for ATN-RNA application in breast cancer (165). ATN-RNA treatment significantly downregulated TN-C in MDA-MB-231 cells and reduced many TN-C-mediated activities for tumor progression, such as cancer cell proliferation and migration. Although, the ATN-RNA approach shows some apparent benefits, systemic delivery of RNAi to the patients remains one of the most difficult challenges in the clinic. We are not aware of any RNAi approaches targeting TN-W in the tumor stroma.

**Table 1:** Strategies for targeting TN-C in cancer treatment.

TN-C	COMPOUND	APPLICATION	CANCER TYPE/MODEL	CLINICAL TRIALS	OUTCOME	REF
**INTERFERENCE RNA**	ATN-RNA (anti TN-C dsRNA)	Injection into the brain after tumor resection	Glioma	11 patients	Significant extension of survival	(162)
	ATN-RNA (anti TN-C dsRNA)	Injection into the brain after tumor resection	Glioma, grade II, III, and IV	46 patients	Increased overal well-being and survival	(163, 164)
	ATN-RNA (anti TN-C dsRNA)	“Proof of concept” in MDA-MB-231 cells	Breast cancer cells	preclinical	Reduced tumor cell proliferation and migration	(165)
**FUNCTION BLOCKING ANTIBODIES**	Single domain nanobodies (Nbs)	“Proof of concept” in KRIB cells	Osteosarcoma cells	preclinical	TN-C function-blocking	(166)
**PEPTIDES**	PL1 (targeting FN-EDB and TN-C-C) loaded with pro-apoptotic payload	10 injections every other day	Xenograft mose model of glioma (U87-MG)	preclinical	Reduced tumor size and prolonged median survival	(167)
	PL3 (targeting TN-C-C and NRP1) loaded with pro-apoptotic payload	10 injections every other day	Xenograft mose model of glioma (U87-MG)	preclinical	Prolonged survival	(168)
	Ft peptide (targeting TN-C and NRP1) loaded with paclitaxel	Intravenously administered every 2 weeks for 3 times	Xenograft mose model of glioma (U87-MG)	preclinical	Prolonged survival	(169)
**APTAMERS**	^99^mTc-TTA1	Tumor imaging and targeted delivery of payload	Xenograft mouse models	preclinical	Specific uptake into tumors (glioma)	(170–172)
	GBI-10	SELEX for TN-C	–	–	Several TN-C-specific sequences were bound	(173)
	^18^F-Fb-TN-C	PET tracer based on a TN-C ssDNA aptamer	Xenograft models (glioma, lung, melanoma cell lines)	preclinical	Tumor-specific uptake with high tumor-background ratio	(174)
	^64^Cu-NOTA-TN-C	PET tracer based on a TN-C ssDNA aptamer	Xenograft models (glioma, lung, melanoma cell lines)	preclinical	Tumor-specific uptake with high tumor-background ratio	(174)
	SMART	Cancer imaging probe	Cell lines *in vitro*	preclinical	Enhanced specificity and signal intensity when compared to “mono” probes	(175)
**ANTIBODY-DRUG-CONJUGATES**	Tenatumomab-^131^I (sigma-tau i.f.r. S.p.A.)	RI	Various solid cancers	Phase I; NCT02602067; terminated	Uptake of drug into the tumor lesion was negligible	
	81C6-^131^I (Neuradiab, Bradmer Pharmaceuticals)	RI after resection followed by systemic chemotherapy	Primary or metastatic brain cancer	Phase I/II	Low toxicity and prolonged survival	(176, 177)
	81C6-^131^I (Neuradiab, Bradmer Pharmaceuticals)	RI combined with temozolomide	Glioma, grade IV	Phase III; NCT00615186; terminated	Delay in site initiation and funding considerations	
	81C6-^131^I (Neuradiab, Bradmer Pharmaceuticals)	RI after resection	Recurrent brain and central nervous system tumors	Phase I; NCT00002753; completed	Unknown	
	81C6-^131^I (Neuradiab, Bradmer Pharmaceuticals)	Bolus injection vs. microinfusion	Primary brain and central nervous system tumors	Phase I/II; NCT00003478; completed	Unknown	
	81C6-^131^I (Neuradiab, Bradmer Pharmaceuticals)	RI after resection	Primary or metastatic brain cancer	Phase I/II; NCT00002752; completed	Unknown	
	81C6-^131^I (Neuradiab, Bradmer Pharmaceuticals)	RI after tumor resection combined with carmustine or irinotecan	Primary brain cancers	Phase I; NCT00003484; completed	Unknown	
	81C6-^131^I (Neuradiab, Bradmer Pharmaceuticals)	Combined with bevacizumab (Avastin)	Glioma, grade IV	Phase II; NCT00906516; unknown	Unknown	
	81C6-^211^At (Bradmer Pharmaceuticals)	RI after resection	Primary or metastatic brain cancer	Phase I/II; NCT00003461; completed	Unknown	
	81C6-^211^At (Bradmer Pharmaceuticals)	RI after resection follwed by systemic chemotherapy	Primary or metastatic brain cancer	Phase I/II	Tolerable toxicity and promising response	(178)
	F16-IL2 (Teleukin, Philogen)	Combined with doxorubicin	Advanced solid tumors, breast cancer	Phase I/II; NCT01131364; terminated	Safe administration	(179)
	F16-IL2 (Teleukin, Philogen)	Combined with paclitaxel	Solid tumors, breast cancer, metastatic melanoma, lung cancer	Phase I/II; NCT01134250; completed	Safe administration/disease stabilization	(180)
	F16-IL2 (Teleukin, Philogen)	Combined with cytarabine	AML, relapse, adult	Phase I; NCT0297032; active	Marked reduction of AML lesions; clinical improval	(181)
	F16-IL2 (Teleukin, Philogen)	Combined with paclitaxel	Merkel Cell Carcinoma	Phase II; NCT02054884; terminated	Terminated due to lack of enrollment	
	F16-IL2 (Teleukin, Philogen)	Combined with anti-CD33 antibody BI 836858	AML relapse after allogeneic stem cell transplantation	Phase I; NCT03207191; completed	Unknown	
	F16-IL2 (Teleukin, Philogen)	Combined with temozolomide	Xenograft mose model of glioma (U87-MG)	preclinical	Complete remission	(182)
	F16-^131^I (Tenarad, Philogen)	RI	Solid tumors, Hodgkin’s lymphoma	Phase I/II; NCT01240720; completed	Partial response/stabilization; tolerable toxicity	(183)
	F16-^124^I (Philogen)	RI	Head and neck cancer	Phase 0	Tumor-specific uptake and good tolerance	(184)
	BC-2-^131^I; BC-4-^131^I	RI	Glioma, recurrent	Phase II; -; completed	Partial stabilization	(185)
	BC-4-biotin + avidin + ^99^Y-biotin	Pre-targeted antibody-guided RI	Glioma	Phase I; -; completed	Stabilization	(186)
**PEPTIDE-ANTIBODY**	iRGD peptide fused to TN-C-C antibody G11	Tail vein injection	Xenograft mose model of glioma (U87-MG)	preclinical	Improved homing to blood vessels, extravasation, and penetration of tumor parenchyma	(187)

Summary of the strategies mentioned in the text for TN-C-targeted approaches in cancer treatment. If available, the NTC identifiers are indicated (www.clinicaltrials.gov). Table was adapted from reference (47). Note that so far there are no clinical trials aimed to target cancer-specific TN-W, TN-R or TN-X. RI, Radioimmunotherapy; AML, Acute Myeloid Leukemia; IL2, Interleukin-2; SELEX, Systematic Evolution of Ligands by Exponential Enrichment; SMART, Simultaneously Multiple Aptamers and RGD Targeting; RGD, arginine-glycine-aspartic acid; PET, Positron Emission Tomography; ds, double-stranded; ss, single-stranded; TN-C, Tenascin-C; TN-C-C, Tenascin-C with extradomain C; FN-EDB, Fibronectin with extradomain B; NRP1, Neuropilin-1; REF, References.

##### 5.2.2.1 Function Blocking Antibodies

In an effort to develop novel and better tools to detect human TN-C, recombinant single-domain nanobodies (Nbs) have been recently raised in the dromedary (166). Nbs possess favorable properties over regular antibodies as they present a high stability, solubility, specificity as well as low immunogenicity. These Nbs recognized the 5^th^ FNIII repeat of TN-C and were able to specifically detect TN-C in formalin-fixed and paraffin-embedded tumor sections. The Nbs also abolished certain pro-tumorigenic functions of TN-C, such as cell adhesion modulation on a FN substratum as well as stromal retention of dendritic cells DC2.4 by CCL21/TN-C. These results promote the newly developed TN-C-specific Nbs as novel molecular tools for detecting TN-C in tumors (diagnostic biomarker) as well as for their potential use as anti-cancer treatment options by blocking the oncogenic functions of TN-C (**Table 1**). Although TN-W-specific single-domain antibodies have been developed in the dromedary as well (188), so far they only have been tested for the specific detection and imaging of TN-W and not for blocking its functions.

##### 5.2.2.2 Affinity Ligands to TN-C and TN-W as Anti-Cancer Strategy

Well-known challenges faced by conventional cytotoxic anti-cancer drugs are their side effects in normal tissues and the barriers in the organism that the drugs need to cross to reach the target site. Specificity is lacking and high drug doses are required for reaching the required local compound concentration. For these reasons, drug-targeting strategies represent promising new avenues for anti-cancer treatment. High-affinity targeting ligands, such as antibodies, peptides, and aptamers can be used that specifically recognize a molecule overexpressed at the target site. By coupling active pharmacological molecules, it should be possible to maximize the therapeutic effects by specific drug delivery. The idea of targeted therapy was initially developed by Ehrlich more than 100 years ago, who proposed to kill pathogens without harming the body [reviewed by (189)]. This simple concept can be and is currently applied to cancer therapy where malignancies must be specifically targeted and eliminated. TN-C, including tumor-specific isoforms, and TN-W represent very promising targets for high-affinity ligands as they are both explicitly enriched in various tumor stroma and as ECM proteins represent quite stable antigens. The benefits in doing so are twofold: TN-C and TN-W are expressed and accessible in the TME (target site), and their levels represent excellent biomarkers for treatment success. As indicated before, available data so far speak in favor of TN-W being superior to TN-C as a tumor biomarker. This holds true both in regard to its tumor-specific expression and to its more significant threshold defining the stage of transformation from normal to malignant tissues as compared to TN-C. However, as it was identified almost 40 years ago, there is much more known about TN-C biology, function, and expression and more TN-C-specific tools have been developed for studying and targeting TN-C in animal models, preclinical, and clinical trials than for TN-W. Indeed, TN-C targeting approaches are actively exploited in *in vivo* models as well as in clinical trials for delivering drugs (e.g., cytokines, radionuclides) specifically to distinct cancer tissues (**Table 1**). Several different tumor-homing agents have been used to target TN-C, including antibody fragments, monoclonal antibodies, peptides, and aptamers. Optimally, the ligand should possess high-binding affinity for TN-C and low immunogenicity. We present a short overview about the most promising approaches, tools and studies, which utilize TN-C as delivery address for anti-cancer payloads.

###### 5.2.2.2.1 Peptides

During the last two years several novel peptide-based approaches for TN-C-targeted anti-cancer therapies have been developed. A peptide PL1 that targets bi-specifically the oncofetal FN isoform (FN-EDB) and a large TN-C isoform (TN-C-C) was identified and shown to home exclusively to the TME as well as to glioblastoma and prostate carcinoma cells in xenografts models (167). When loaded with a pro-apoptotic peptide, the PL1-targeted model nanocarrier was able to shrink glioblastoma in mice and significantly increase their survival. The same authors also developed a specific, octameric PL3 peptide targeting TN-C and cell- and tissue-penetration receptor NRP1. Promisingly, nanoparticles containing PL3-targeted anticancer payloads proved to be very useful in detection and systemic therapy in prostate carcinoma, glioma, and melanoma cells and in the corresponding xenograft mouse models, as the animal significantly survived for longer periods (168). A similar approach was applied by Kang et al. They also used the combination of TN-C and NRP1 as homing addresses for targeting the tumor ECM and neovasculature, respectively. When loaded with paclitaxel, the synergistic dual-targeting nanocarrier showed higher cytotoxic effects and apoptosis rate compared with mono-targeting approaches in glioma models (169).

###### 5.2.2.2.2 Aptamers

Aptamers are short single-stranded oligonucleotides (DNA or RNA) and represent an alternative approach for targeting approaches in tumors. They possess high affinity and selectivity for a specific target, while there is low immunogenicity, amenability to modifications and conjugations, as well as favorable pharmacokinetics. Several TN-C-specific aptamers have been developed that can be used as imaging tools [^99^mTc-TTA1 (170–172) and GBI-10 aptamers (173)] and PET tracers [^18^F-Fb-TN-C and ^64^Cu-NOTA-TN-C aptamers (174)] to localize and detect tumor-specific TN-C expression allowing the planning of personalized treatment strategies as well as tumor monitoring. Aptamers are usually taken up by a variety of solid tumors and are rapidly cleared from the blood and other nontarget tissues, which add to their beneficial properties. A multimodal nanoparticle-based Simultaneously Multiple Aptamers and RGD Targeting (SMART) cancer imaging probe, which is able to simultaneously target nucleolin (AS1411 aptamer), integrin a_v_β3 (RGD), and TN-C (TTA1 aptamer) hes been described by Ko et al. (175). This probe showed enhanced tumor targeting efficacy and a better signal-to-noise ratio compared to mono-biomarker targeting approaches in various cancers (175). Moreover, TN-C targeting aptamers can also be used as vehicles for delivering radioisotopes or chemical agents to cancerous tissues and present novel potential therapeutic applications. So far, clinical trials with aptamers targeting TN-W have not been reported.

###### 5.2.2.2.3 Antibodies

Antibody-drug-conjugate (ADC) technology aims to home a toxic agent specifically into the tumor using target-specific antibodies. Several ADCs have shown impressive results in treating cancers, which resulted in the approval of 11 different ADCs by the FDA that target hematologic as well as solid malignancies (190). Four crucial features might decide the success or failure of ADC applications: (i) specific tumor targeting; (ii) the property of the antibody itself; (iii) the nature of the cytotoxic payload; and (iv) the method used for linking the payload to the antibody. As described, TN-C and especially tumor-specific large TN-C isoforms (as well as TN-W) represent interesting tumor marker candidates for ADCs. Indeed, several monoclonal antibodies recognizing isoforms of TN-C are in preclinical and clinical stages. For instance, ^131^I- and ^211^At-labeled chimeric 81C6 monoclonal antibody (Neuradiab recognizing TN-C-C/D) as monotreatment or in combination with other compounds for the treatment of patients with non-Hodgkin lymphoma, with recurrent glioma, with primary or metastatic brain cancer showed encouraging initial results with relatively low toxicity (176–178). Additional TN-C-specific antibodies have been tested in recurrent malignant glioma patients (antibodies BC-2-^131^I, BC-4-^131^I; clinical phase II) and in anaplastic astrocytoma and glioblastoma patients (BC4-biotin + avidin + ^99^Y-biotin; clinical phase I) and revealed disease stabilization with partial remission (185, 186). Another promising tool consisting of a fully humanized antibody F16 recognizing the variable FNIII domain A1 of TN-C has been developed by Philogen (191). In the small immunoprotein format (SIP), F16 was able to recognize TN-C in several different cancers such as glioblastoma, lymphoma, melanoma, head and neck, and renal cell carcinoma (182, 192–196). The promising biodistribution of F16 prompted its testing for radionuclide as well as cytokine/chemokine combination therapy. F16-^124^I has been applied in four head and neck cancer patients resulting in a tumor-specific signal in all patients within 24 hours (184). Phase I/II clinical dose finding and efficacy trials (www.clinicaltrials.gov; NCT01240720) have been performed with F16-^131^I (Tenarad) in eight Hodgkin lymphoma patients (183). Treatment of the patients with F16-^131^I did not cause any severe toxicity problems and was effective in providing clinical benefit in the majority of the patients. Apart from radiotherapies, F16SIP is currently also evaluated for its potential as combinatory treatment option when coupled to IL2, which elicits an immune response that is able to kill cancer cells. So far, F16-IL2 (Teleukin, Philogen) has been applied in combination with paclitaxel for breast cancer, melanoma, non-small lung cancer [NCT01134250 (180)] as well as in metastatic Merkel cell carcinoma patients (NCT02054884), together with low doses of cytarabine in AML patients with advanced disease (NCT02957032 (181), and simultaneously with doxorubicin in advanced solid tumor and metastatic breast cancer patients (NCT01131364). Initial results for the studies using F16-IL2 are promising as it can be safely and repeatedly administered to patients with lung cancer in combination with paclitaxel (180) and to patients with different solid and metastatic breast cancer together with doxorubicin (179). The strong safety profile and the appearance of early signs of clinical activity of F16-IL2 warrant further studies.

Tumor-homing, extravasation, and penetration of cancer drugs can be improved by conjugation to tumor penetrating iRGD peptides, which is a widely used approach (197–199). Several clinical trials are ongoing in pancreas, colon, and digestive cancers testing the effect of iRGD alone [NCT05052567 (not yet recruiting)] in combination with anti-cancer drugs nabpaclitaxel and gemcitabine [NCT03517176 (completed), NCT05042128 (not yet recruiting)], or with panitumumab [NCT05121038 (recruiting)]. Recently, a study analyzed the effect of genetic fusion of the iRGD peptide to recombinant anti-TN-C-C single-chain antibody clone G11 (58) in U87-MG glioma tumor models compared to the parental antibody (187). Comparative biodistribution studies revealed improved homing to blood vessels, extravasation, and tumor penetration in the presence of the iRGD peptide (187). Such an approach using a tumor-penetrating peptide-functionalized TN-C antibody could be more efficient in delivering therapeutic agents into solid tumor lesions and may develop into a useful strategy for affinity targeting of solid tumors.

## 6 Concluding Remarks

Tenascins are a family of matricellular proteins that show intriguing expression patterns. During development they are expressed widely in various tissues reflecting their important role in organ and tissue development. In most healthy adult tissues their levels of expression are greatly diminished. Upon disturbance of tissue homeostasis, which often accompanies the initiation of a tumor (200), TN-C and TN-W are sharply upregulated in the TME. There, they make use of their multifunctionality and interact with several other ECM proteins and cell surface receptors, eliciting signaling pathways that drive tumor progression (201).

In the embryo and in the TME tenascins are part of a complex microenvironment with many other ECM molecules and growth factors. With these many potential interaction partners available, tenascins have multiple possibilities to influence cell fate. This enormous complexity makes the study of the real *in vivo* effects and functions of tenascins in physiological as well in pathological conditions challenging. Can we really study tenascin function in a 2D tissue culture dish? Is the use of 3D conditions or Matrigel of any advantage to determine the *in vivo* function of these molecules? What all these study models have in common is the limitation that none can fully recapitulate the microenvironment as it exists *in vivo*. Still, the proper understanding and a thorough characterization of the tenascin members is of utmost importance. Even if the study conditions do not fully reflect the complexity of the *in vivo* situation, much has been and still can be learned from these experimental approaches. This is especially true for TN-C and TN-W, which are both prominently over-expressed in tumor stroma. The many pre-clinical and clinical studies trying to harness their tumor-specific expression for diagnostic and therapeutic use could not have been done without understanding the basics about TN-C and TN-W biology.

Fundamental to the study of TN-C and TN-W in clinical settings is the availability of excellent antibodies for their detection. For both proteins, outstanding antibodies have been raised (88, 147, 188), and more recent efforts generated TN-C-specific Nbs (166), which might be highly useful for future studies and clinical trials. Unfortunately, research on TN-W lags behind the research focused on its older brother, TN-C. In contrast to TN-C, TN-W function has not been thoroughly elucidated yet and there still remains a lot to be learned about the role of TN-W in tumors, which might clarify why it is expressed at a high level in many cancers. A better understanding of TN-W function and regulation, as well as its interactions with cellular receptors in tumors, might also provide the basis for more attempts in using it as a cancer biomarker or therapeutic target. This, of course, raises the questions about the importance of TN-C and TN-W in tumor ECM. Is it feasible to expect better cancer management or a normalization of the TME by targeting one specific aberrantly expressed ECM protein present in the tumor stroma, such as TN-C, while we know that the TME consists of a 3D meshwork with seemingly countless components? Perhaps after targeting TN-C in tumors stroma, TN-W can compensate for its absence and, therefore, a positive treatment effect is only short-lived or only achieved if both tenascins are targeted. The clinical trials with drug-delivery vehicles targeting TN-C produced some encouraging results in their initial phases (see above). Certainly, it is worth continuing this path of research and making use of their highly regulated expression in tumors. In this regard, TN-W might also represent an interesting and promising tumor-specific homing address for drug delivery: (i) TN-W is often expressed around blood vessels in the tumor stroma, which are often leaky making antibody administration feasible; (ii) TN-W is not known to be upregulated in pathologies other than cancers; and (iii) TN-W is generally not expressed in normal tissues with some few exceptions. However, we are currently not aware of such attempts. It also remains to be understood why among patients suffering from the same type of tumor, TN-W and TN-C expression is very heterogenous. Some patients display very high levels of the tenascins, while other patients only show modest expression [see for example (88)]. Which biochemical, biophysical or clinical properties dictate their regulation of expression in cancer?

Herein, we attempted to summarize some of the knowledge about the tenascin family of ECM proteins in health and cancer. Although we have learned much over the last 40 years, there is still much to be discovered. We are still far from being able to depict the full picture of the specific roles of each of the tenascin members and how they are incorporated into complex ECM networks, and how they are precisely regulated in health and disease. This is true for all four members. As of today, TN-C and TN-W are the most promising family members to be exploited for anti-cancer treatment. However, this does not diminish the potential roles that TN-R and TN-X might play in tumorigenesis. Clearly, more work is required to address and recognize the pleiotropic roles of all tenascin members in physiological as well as in pathological conditions. Only a broader understanding of tenascin biology might give us the chance to use them as exploitable matricellular proteins for cancer diagnosis and monitoring as well as for anti-cancer therapies.

## Author Contributions

RT wrote the first draft of the abstract and section 2, MD wrote the first draft of the remaining sections. Both authors contributed to the figures and the final draft. All authors contributed to the article and approved the submitted version.

## Funding

Open access publication fees have been paid by the University of Bern, Switzerland.

## Conflict of Interest

The authors declare that the research was conducted in the absence of any commercial or financial relationships that could be construed as a potential conflict of interest.

## Publisher’s Note

All claims expressed in this article are solely those of the authors and do not necessarily represent those of their affiliated organizations, or those of the publisher, the editors and the reviewers. Any product that may be evaluated in this article, or claim that may be made by its manufacturer, is not guaranteed or endorsed by the publisher.
